# Trial forge – working together to make trials more efficient

**DOI:** 10.1186/1745-6215-16-S2-P231

**Published:** 2015-11-16

**Authors:** Shaun Treweek

**Affiliations:** 1 University of Aberdeen, Aberdeen, UK

## 

Randomised trials are at the heart of evidence-based healthcare but the methods and infrastructure used to conduct these complex studies are a largely evidence-free zone. Trial Forge (http://www.trialforge.org) is an initiative that aims to increase the evidence base for trial decision-making and in doing so, improve trial efficiency.

That trials are inefficient is not in doubt. Inefficiencies around research questions may produce trials that run well but do not increase the evidence base: think of ‘me-too’ non-inferiority trials. Many trials struggle with recruitment and retention, or collect data that go unpublished. This talk will describe the Trial Forge trial pathway, developed at a workshop of trialists in 2014, that maps out key trial processes. This pathway provides targets for systematic evidence collation and helps to identify evidence gaps (e.g. around the use of multimedia during trial consent, or how to reduce attrition from face-to-face trial measurement visits). Trial Forge aims to directly facilitate collaboration between research groups and other stakeholders to address these evidence gaps.

The talk will present examples of Trial Forge at work, including work on how time is allocated to collecting primary and secondary outcomes, how public involvement in research design can be made easier and how to improve site selection. These are first steps in what aims to become a global approach to improving trial efficiency. Collaboration is key and more likely to lead to step-changes in trial efficiency than a series of high quality, but uncoordinated, methodological studies.

